# Improved Expression of SARS-CoV-2 Spike RBD Using the Insect Cell-Baculovirus System

**DOI:** 10.3390/v14122794

**Published:** 2022-12-15

**Authors:** Joaquín Poodts, Ignacio Smith, Joaquín Manuel Birenbaum, María Sol Rodriguez, Luciano Montero, Federico Javier Wolman, Juan Ignacio Marfía, Silvina Noemí Valdez, Leonardo Gabriel Alonso, Alexandra Marisa Targovnik, María Victoria Miranda

**Affiliations:** 1 Cátedra de Biotecnología, Departamento de Microbiología, Inmunología, Biotecnología y Genética, Facultad de Farmacia y Bioquímica, Universidad de Buenos Aires (UBA), Junín 956, Buenos Aires 1113, Argentina; 2 Instituto de Nanobiotecnología (NANOBIOTEC), Universidad de Buenos Aires (UBA), Consejo Nacional de Investigaciones Científicas y Técnicas (CONICET), Junín 956, Buenos Aires 1113, Argentina; 3 Cátedra de Inmunología, Departamento de Microbiología, Inmunología, Biotecnología y Genética, Facultad de Farmacia y Bioquímica, Universidad de Buenos Aires (UBA), Junín 956, Buenos Aires 1113, Argentina; 4 Instituto de Estudios de la Inmunidad Humoral “Prof. Ricardo A. Margni” (IDEHU), Universidad de Buenos Aires (UBA), Consejo Nacional de Investigaciones Científicas y Técnicas (CONICET), Junín 956, Buenos Aires 1113, Argentina

**Keywords:** COVID-19, SARS-CoV-2, RBD, baculovirus, insect cells, Sf9, expression

## Abstract

Insect cell-baculovirus expression vector system is one of the most established platforms to produce biological products, and it plays a fundamental role in the context of COVID-19 emergency, providing recombinant proteins for treatment, diagnosis, and prevention. SARS-CoV-2 infection is mediated by the interaction of the spike glycoprotein trimer via its receptor-binding domain (RBD) with the host’s cellular receptor. As RBD is required for many applications, in the context of pandemic it is important to meet the challenge of producing a high amount of recombinant RBD (rRBD). For this reason, in the present study, we developed a process based on Sf9 insect cells to improve rRBD yield. rRBD was recovered from the supernatant of infected cells and easily purified by metal ion affinity chromatography, with a yield of 82% and purity higher than 95%. Expressed under a novel chimeric promoter (*polh-pSeL*), the yield of rRBD after purification was 21.1 ± 3.7 mg/L, which is the highest performance described in Sf9 cell lines. Finally, rRBD was successfully used in an assay to detect specific antibodies in COVID-19 serum samples. The efficient strategy herein described has the potential to produce high-quality rRBD in Sf9 cell line for diagnostic purpose.

## 1. Introduction

In December 2019, a new coronavirus named severe acute respiratory syndrome coronavirus 2 (SARS-CoV-2) was identified as the etiologic agent of a new respiratory disease called coronavirus disease 2019 (COVID-19). Since the initial outbreak in China, COVID-19 has spread globally becoming one of the most challenging episodes in the history of modern public health. By November 2022, SARS-CoV-2 had infected more than 600 million people and caused more than 6 million deaths worldwide as reported by the World Health Organization [1]. 

The SARS-CoV-2 is an envelope virus containing a positive-strand, non-segmented RNA genome that encodes 29 proteins, including 25 putative non-structural and accessory proteins, and four structural proteins [2]. Among its structural proteins, the spike glycoprotein (S) is the main antigenic component involved in host cell recognition and viral entry [3]. The S protein belongs to the type I viral fusion protein and comprises two functional subunits, S1 and S2 [4]. In particular, S1 contains the Receptor-Binding Domain (RBD) in the SARS-CoV-2 S protein, which binds strongly to angiotensin-converting enzyme 2 (ACE2) on the host cell membrane and constitutes the main target of neutralizing antibodies [5]. In addition to the soluble spike trimer, RBD is the clear target for therapeutic interventions such as vaccines or monoclonal antibodies, and also for use in serology assays [6,7]. Given that coronavirus genomes are constantly subject to genomic rearrangement and point mutation favoring the emergence of SARS-CoV-2 variant, it is crucial to have biotechnological platforms that can quickly adapt to the production of recombinant RBD (rRBD) from SARS-CoV-2 variants [8]. For instance, one of the recent variants (Omicron) possesses a large number of mutations: more than 30 in the S protein, 15 of which are contained in RBD [9]. 

In recent years, the insect cell-baculovirus expression vector system (IC-BEVS) has been extensively used to produce viral structural proteins. The IC-BEVS is one of the most established platforms to produce biological products in order to provide solutions to emerging human disease [10,11]. Moreover, there are already numerous products on the market produced in IC-BEVS for the treatment and prevention of human disease. The platform fulfilled a fundamental role in the COVID-19 emergency. In this regard, the recently marketed Novavax COVID-19 vaccine was produced in Sf9 cell line [12]. This system is a more cost-effective and scalable platform to produce eukaryotic recombinant proteins than traditional mammalian cell-based methods [13]. Moreover, it is completely safe, poses no risk to the operator, and provides proper protein folding, post-translational modifications, and strong promoters, which renders it an attractive system [14]. The insect cell lines traditionally used for producing recombinant proteins that are susceptible to the prototype baculovirus species *Autographa californica multiple nucleopolhyedrovirus* (AcMNPV) are High Five™ derived from *Trichoplusia ni* and Sf9 derived from *Spodoptera frugiperda*. It was reported that the High Five™ cell line is considered a better host to produce recombinant proteins in BEVS than Sf9 insect cell lines [12]. For this reason, most scientific reports use the High Five™ line as a host to produce rRBD, reaching a maximum yield of 6.5 mg/L [15,16,17]. It was demonstrated that High Five™ produces glycoproteins with core alfa 1,3-fucose structure with allergenic potential when used in humans, which does not occur with the Sf9 insect cell line [18]. So far, the only study reporting on rRBD expression under the traditional polyhedrin (*polh*) promoter in IC-BEVS using Sf9 cell as a host achieved poor yield (0.8 mg/L culture) [19]. The rRBD production process in Sf9 cells has not been optimized and could be improved by using alternative promoters to the traditional ones, such us *polh* and *p10*. Recently, the development of new chimeric promoters, such as *polh-pSeL*, has made it possible to significantly increase the yield achieved in Sf9 insect cells [20,21].

In this report, we describe a novel process to produce a high amount of rRBD in Sf9 insect cell line using BEVS. This high-quality and pure recombinant antigen is useful for the development of COVID-19 reagents.

## 2. Materials and Methods

### 2.1. Construction of pFBD-polh-pSeL-X Baculovirus Shuttle Vector

The *polh* promoter from pFastBac™ Dual (Thermo Fisher Scientific, Waltham, MA, USA) vector was replaced with the chimera promoter *polh-pSeL* developed by the Dr. Salvador Herrero’s laboratory at Universitat de València [20,21]. For this purpose, the *polh-pSel* promoter fragment was obtained from the *polh-pSeL*-GFP plasmid by digestion with *BstZ17*I and *EcoR*I enzymes. The fragment was then inserted into the *BstZ17*I and *EcoR*I sites of the pFastBac™ Dual (Thermo Fisher Scientific), generating the pFBD-*polh-pSeL*-X vector. In addition, the enhanced green fluorescent protein (EGFP) cDNA was cloned into *Sma*I and *Nco*I sites under the *p10* promoter, as previously described [21].

### 2.2. RBD cDNA Sequence and Cloning into pFBD-polh-pSeL-X Baculovirus Shuttle Vector

The detailed procedure for constructing the baculovirus is shown in Supplementary Figure S1. The full-length spike cDNA sequence from SARS-CoV-2 was previously synthetized and codon optimized for insect cell expression by GenScript (Piscataway, NJ, USA). The sequence was cloned into pFastBac™ Dual vector (Thermo Fisher Scientific) and codified for spike protein Wuhan-Hu-1 isolated (GenBank accession no. QHD43416.1) [7]. Using this vector as a template, the RBD cDNA sequence—composed of 955 bp to 1623 pb corresponding to amino acid residues 319–541 of the S protein—was amplified by PCR using two specific primers. These primers added *BamH*I and *EcoR*I restriction sites and six histidine residues (His-tag): 5′-CGCGGATCCAGGGTGCAGCCTACCGAATC-3′ (primer sense; *BamH*I site underlined) and 5′-CCGGAATTCTTA**GTGGTGGTGGTGATGATG**GAAGTTCACGCACTTGTTCTTG-3′ (primer antisense, *EcoRI* site underlined and His-tag codon in bold). The PCR conditions (50 μL final volume) were as follows: 200 nM of each primer, 1 × PFU buffer, 0.3 mM of each dNTP, and 2.5 U PFU polymerase (Promega, Madison, WI, USA). The PCR program was 95 °C for 6 min, 95 °C for 30 s, 56 °C for 30 s, and 72 °C for 1 min/kb × 30 cycles. An additional extension step of 72 °C for 5 min was then applied. After the reaction, the PCR product was purified by using the PCR WizardTM SV gel and PCR Clean-up System (Promega). The rRBD sequence fused to the His-tag sequence was then cloned using the *BamH*I and *EcoR*I sites of the pAcGP67-B vector (BD Biosciences, San Diego, CA, USA) to construct pAcGP67-rRBD, which contained the nucleotide sequence that encodes the baculoviral glycoprotein 64 leader peptides (GP64; syn.: GP67). Using pAcGP67-rRBD as a template, the rRBD sequence fused with the GP64 signal peptide (gprRBD) and His-tag was amplified following the protocol described above. This was done by PCR using two specific primers, which added the *EcoR*I restriction sites: 5′-CCGGAATTCATGCTACTAGTAAATCAGTCAC-3′ (primer sense, *EcoR*I site underlined) and 5′-CCGGAATTCTTA**GTGGTGGTGGTGATGATG**GAAGTTCACGCACTTGTTCTTG-3′ (primers antisense, *EcoRI* site underlined). Finally, the gprRBD cassette was cloned into the pFBD-*polh-pSeL*-X vector under the *polh-pSeL* promoter using the *EcoR*I site to construct the pFBD-*polh-pSeL*-gprRBD. All the DNA constructs were verified by Sanger sequencing.

### 2.3. Insect Cell Culture

The *S. frugiperda* (Sf9) insect cell line (Thermo Fisher Scientific) was maintained in suspension cultures in a sterile Erlenmeyer flask and grown in Sf-900™ III SFM medium (Thermo Fisher Scientific) supplemented with 1% (*v*/*v*) antibiotic-antimycotic solution (Thermo Fisher Scientific) at 27 °C under continuous shaking at 100 rpm. Additionally, the suspension volume did not exceed 10% of the total volume of the Erlenmeyer flask.

### 2.4. Virus Production

The recombinant baculoviruses were obtained by using the Bac-to-Bac^®^ baculovirus expression system (Thermo Fisher Scientific), following the manufacturer´s instructions. The pFBD-*polh-pSeL*-gprRBD vector was transformed into chemically competent *E. coli* DH10Bac™ strain (Thermo Fisher Scientific) by heat shock to generate the recombinant bacmid by transposition. The bacmids were then purified and used to transfect one million Sf9 cells by using Cellfectin II Reagent (Thermo Fisher Scientific). After 4-day incubation at 27 °C, the cell culture supernatant was collected and centrifuged at 500× *g* for 10 min. The transfection efficiency was determined by measuring EGFP expression by fluorescence under UV light. Following three amplification steps, the virus titer was determined by a plaque assay [22]. The amplified virus stock was used for producing the rRBD in further experiments.

### 2.5. Insect Cell Infection

For the rRBD expression assay, independent Sf9 (serial passage 30) suspension cultures in log-phase at a cell density of 4 × 10^7^ cells in 20 mL (2 × 10^6^ cell/mL) were infected with *Acpolh-pSeL*-gprRBD multiplicity of infection (MOI) of 1 [21]. The infected suspension culture was incubated in an orbital shaker at 100 rpm in the dark at 27 °C for 4 days. To study the expression at different days post-infection (dpi), samples of 1 mL were collected each day. The culture supernatant was separated from the cell by centrifugation at 500× *g* for 10 min. The pellet and the supernatant were stored at −20 °C until further experiments. An Sf9 suspension culture infected with baculovirus *AcMNPVHRPC*—previously constructed in our laboratory—was also included as a control [23].

### 2.6. Total Protein Measurement

Total protein concentration was determined by following the Bradford micro-assay protocol [24] with bovine serum albumin (BSA) as the standard, using the Quick Start™ Bradford reagent (BioRad, Hercules, CA, USA). 

### 2.7. Electrophoretic Analysis 

The protein samples were resolved by SDS-PAGE on 12.5% or 15% polyacrylamide gels. The samples were heated at 100 °C for 5 min in sample buffer [125 mM Tris/HCl, pH 6.8, 4% (*w*/*v*) SDS, 20% (*w*/*v*) glycerol, 0.01% (*w*/*v*) bromophenol blue, 10% (*v*/*v*) 2-mercaptoethanol]. Samples were also prepared under non-reducing conditions by not incorporating 2-mercaptoethanol into the sample buffer. The resulting gels were stained with Coomassie Blue R-250. For western blot analysis, the gels were transferred onto a nitrocellulose membrane (GE Healthcare, Chicago, CA, USA), and rRBD was detected using a mouse anti-His antibody (Thermo Fisher Scientific) in a 1/3000 dilution as the primary antibody and a goat anti-mouse immunoglobulin conjugated with Horseradish Peroxidase (HRP) as the secondary antibody (Jackson ImmunoResearch Laboratories Inc, West Grove, PA, USA). Alternatively, rRBD was detected using an equine polyclonal anti-S antibody serum, previously produced [25] in a 1/5000 dilution as the primary antibody, and a mouse anti-equine immunoglobulin conjugated with HRP as the secondary antibody (Sigma-Aldrich, St. Louis, MO, USA). Protein bands were detected using an enhanced chemiluminescent substrate (ECL; Thermo Fisher Scientific) and a C-Digit blot scanner (LI-COR, Bad Homburg, Germany). For image processing, the SDS-PAGE gels and western blot were scanned and then analyzed with the ImageJ version 1.51k software (National Institute of Health, Bethesda, MD, USA). The amount and purity of rRBD were assessed by densitometric analysis of the band intensities from the SDS-PAGE and Bradford assay using BSA as the standard. The results were expressed as the mean ± standard deviation of at least three determinations.

### 2.8. rRBD Purification by Immobilized Metal Ion Affinity Chromatography (IMAC)

The sample from insect cell culture was clarified by centrifugation (5000× *g*, 10 min, 4 °C) and buffer exchanged by tangential flow filtration (TFF) using a MasterFlex peristaltic pump (Cole-Parmer, Vernon Hills, IL, USA), and an ultrafiltration module Pellicon XL with Biomax 10 kDa Membrane (Merck, Darmstadt, Germany). Briefly, the clarified supernatant was concentrated 2× and diafiltered with 5 vol of equilibration buffer (20 mM phosphate buffer, pH 8.0, 300 mM NaCl, 20 mM imidazole). The samples were then loaded in a column (11 mm internal diameter) packed with 3 mL Nuvia IMAC Ni-NTA Resin (BioRad) connected to an AKTA Purifier chromatography system (Cytiva, Marloroughm, MA, USA). Following a washing step with 80 mM imidazole, an elution step was performed by increasing the imidazole concentration to 500 mM. The linear flow rate was 2.1 cm/min. Protein separation was monitored by absorbance at 280 nm. All fractions were collected and analyzed by SDS-PAGE and western blot. 

### 2.9. MALDI-TOF Mass Spectrometry

MALDI-TOF MS spectra were recorded on a 4700 Proteomics Analyzer Instrument (Applied Biosystems, Foster City, CA, USA). Samples of IMAC-purified rRBD were loaded with sinapinic acid as the matrix in 30% (*v*/*v*) acetonitrile (ACN) and 0.1% (*v*/*v*) trifluoroacetic acid (TFA) in H_2_O onto a stainless-steel target.

### 2.10. Glycosylation Assay

The purified rRBD was subjected to N-glycosidase F (Roche, Mannheim, Germany) digestion. For this purpose, 20 μg of protein was mixed with denaturing buffer 10× (2.5% SDS, 0.4 M DTT) and H_2_O to a final volume of 10 μL. After sample heating at 100 °C for 10 min, the reaction buffer 10× [0.5 M sodium phosphate buffer, pH 7.5, 10% (*v*/*v*) NP-40], inhibitor cocktail protease, 3 U N-glycosidase F, and H_2_O were added to a final volume of 20 μL. As a control, 20 μg of denatured protein was incubated in the reaction buffer without the enzyme. After incubation at 37 °C for 16 h, the sample was analyzed by SDS-PAGE and western blot, as described above. 

### 2.11. Reverse-Phase-High Performance Liquid Chromatography (RP-HPLC)

Eluted fraction from IMAC was centrifuged for 10 min at 12,000× *g* and injected into RP-HPLC (Shimadzu, Japan) to evaluate the purity level of rRBD using a Bio-Basic C4 column (Thermo Fisher Scientific). The chromatographic run was monitored at 220 nm and 280 nm and the flow used was 1 mL/min. Mobile phase A was 0.1% (*v*/*v*) TFA in H_2_O and mobile phase B was 0.1% (*v*/*v*) TFA in ACN. The chromatogram gradient was performed as 0 to 2.5 min holding 20% B, 2.5 to 25 min from 20% to 65% B, 25 to 30 min holding 65% B, 30 to 31 min from 65% to 20% B, and 31 to 40 min holding 20% B.

### 2.12. Size Exclusion Chromatography (SEC)

The oligomerization state of the rRBD protein was evaluated by SEC. Elution fractions from IMAC containing purified rRBD were collected and concentrated, and the buffer was exchanged and loaded on a Superdex 200 increase 10/300 (Cytiva) equilibrated in 100 mM sodium phosphate buffer pH 7.4 and 150 mM NaCl. The elution peaks were collected, and the rRBD was developed by SDS-PAGE and western blot, as described previously. 

### 2.13. Assessment of rRBD Immunoreactivity

#### 2.13.1. Serum Collection

Control serum/plasma (n = 28) was obtained from healthy individuals before the outbreak of SARS-CoV-2. COVID-19 patient serum/plasma samples were collected from a total of 30 COVID-19 cases confirmed to be infected with SARS-CoV-2 by real-time reverse transcription-polymerase chain reaction (rRT-PCR) on samples from the respiratory tract. These patient samples were IgG-positive for SARS-CoV-2 by COVIDAR IgG ELISA test (Laboratorio Lemos S.R.L., Buenos Aires, Argentina). The samples were provided by the Biobank of Infectious Diseases (BBEI, for its acronym in Spanish) of the Institute for Biomedical Research on Retroviruses and AIDS (INBIRS, for its acronym in Spanish). Sample collection and protocols were approved by the Ethics Committee of BBEI-INBIRS and the Ethics Committee on Clinical Research of the School of Pharmacy and Biochemistry, University of Buenos Aires. All subjects were informed about the purpose of the study, and they signed consent for study participation.

#### 2.13.2. Bridge Enzyme-Linked Immunosorbent Assay (b-ELISA) Using rRBD as Coating Antigen

The rRBD immunoreactivity was evaluated using a bridge enzyme-linked immunosorbent assay previously developed by Trabucchi et al. [25] using purified rRBD instead of S as coating antigen. Briefly, polystyrene microplates (Maxisorp, NUNC, Roskilde, Denmark) were coated overnight at 4 °C with 0.5 μg/mL of purified rRBD per well, washed three times with PBS (1.5 mM KH_2_PO_4_, 8.1 mM Na_2_HPO_4_, 140 mM NaCl, 2.7 mM KCl, pH 7.4), blocked for 1 h with 200 μL of blocking buffer [3% (*w*/*v*) skim milk in PBS], and washed six times with PBS-T (PBS-0.05% Tween 20). Serum/plasma samples were added in duplicate to the coated microplates and incubated for 20 min. Plates were then washed with PBS-T six times, and 50 ng of S protein-biotin per well was added. After another 20 min of incubation, plates were washed with PBS-T six times and bound S protein-biotin was detected by the addition of Streptavidin-HRP (Jackson ImmunoResearch Laboratories Inc.) diluted 1/300. After 20 min of incubation at 37 °C, microplates were washed with PBS-T five times plus one final washing step with 200 μL of PBS; 3,3′,5,5′-tetramethyl-benzidine/H_2_O_2_ (Single Component TMB Peroxidase EIA Substrate Kit, BioRad) was added, and plates were incubated for 15 min in the dark. The color reaction was stopped with 4 N H_2_SO_4_. The oxidized substrate was measured at 450 nm with an ELISA plate reader MultiskanFC (Thermo Fisher Scientific). The blank control was made by replacing serum/plasma samples with PBS-MT [3% (*w*/*v*) skim milk, in PBS-T]. The positive control of the assay was the hyperimmune equine serum anti-S protein. Results were calculated as specific absorbance (A = the mean of each sample minus the mean of the blank control) and expressed as Standard Deviation score (SDs). SDs = (A − Ac)/SDc, where Ac is the mean specific absorbance from pre-pandemic control samples (approximately 20 normal control sera in each assay), and SDc is the corresponding standard deviation between measurements for those control samples. The cut-off value of the assay was set at SDs = 5.0. Statistical significance was evaluated using unpaired-samples Student t-test with Welch correction. Calculations were performed using GraphPad Prism software version 6.01 (San Diego, CA, USA). A *p* value < 0.001 was considered statistically significant.

## 3. Results and Discussion

### 3.1. Generation of the Recombinant Baculovirus Acpolh-pSeL-gprRBD

**Figure 1:**
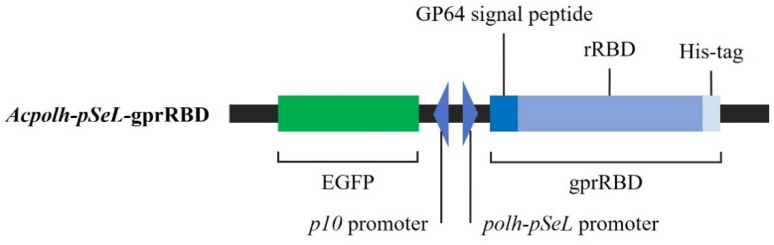
Recombinant baculovirus (*Acpolh-pSeL*-gprRBD) for the expression of rRBD under the *polh-pSeL* promoter. GP64: viral secretion signal GP64; His-tag: six histidine tag. rRBD: SARS-CoV-2 receptor binding domain sequence (Wuhan-Hu-1 isolate) optimized for insects.

The RBD sequence fused to the GP64 viral signal peptide, and a His-tag (gprRBD) was cloned under the control of the *polh-pSeL* promoter to obtain the pFBD-*polh-pSeL*-gprRBD. The GP64 signal peptide ensures post-translational modification and targets the recombinant protein for secretion, whereas the addition of a C-terminal His-tag facilitates the purification step of the rRBD by IMAC. The *polh-pSeL* promoter developed by Martinez-Solís [20] is a strong chimera promoter that combines the *polh* traditional promoter with a fragment of 120 pb belonging to the promoter of *Spodoptera exigua* multiple polyhedrovirus (*pSeL*). All these promoters naturally drive the expression of structural protein, and their combination generally results in an additive effect on the expression level [18,20]. Recently, we demonstrated the power of this promoter to enhance the production of structural viral proteins in the baculovirus-Sf9 insect cell system [21]. Based on these results, the promoter *polh-pSeL* was used for rRBD production. Another characteristic of the expression vector employed in this study is the EGFP sequence cloned under the *p10* promoter used as a reporter gene to allow us to visualize the viral infection. Plasmids were used to produce the corresponding bacmid. Transfection of the bacmids and amplification in Sf9 cells allowed us to obtain the *Acpolh-pSeL*-gprRBD virus (Figure 1).

### 3.2. rRBD Expression in Sf9 Insect Cells

**Figure 2:**
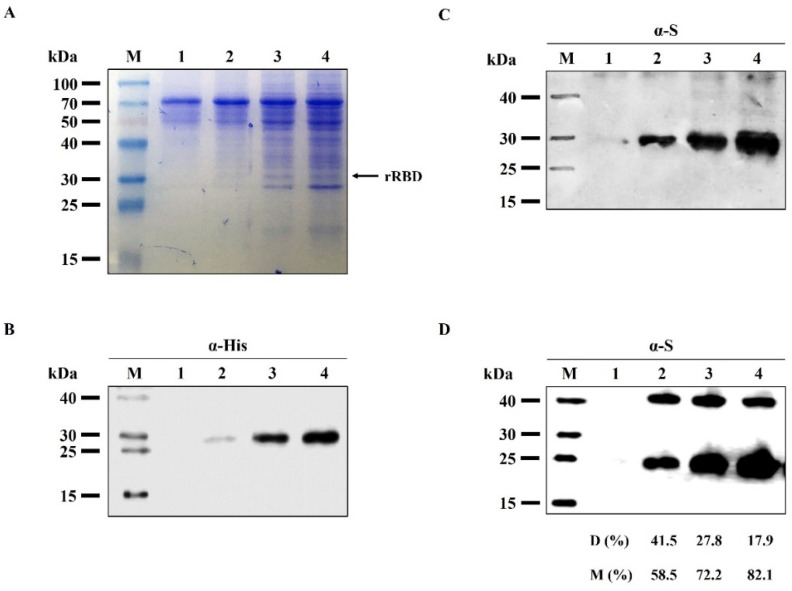
Analysis of rRBD in supernatants by SDS-PAGE under reducing (**A**–**C**) and non-reducing (**D**) conditions followed by western blot developed with anti-His (α-His) and anti-S (α-S) antibodies. Sf9 cells were infected with *Acpolh-pSeL*-gprRBD. At different days post-infection, the culture medium was harvested and analyzed. Lanes: 1–4, culture supernatant from 1–4 days post-infection; M, protein marker; D (%): ratio of dimer to total rRBD expressed as determined by densitometry analysis. M (%): ratio of monomer to total rRBD expressed as determined by densitometry analysis.

To determine rRBD expression, suspension culture of Sf9 cells were infected with *Acpolh-pSeL*-gprRBD at MOI 1. The expression in cell culture supernatant was evaluated by western blot at 4 dpi in order to avoid cell protein contamination in the purification step (Figure 2). The soluble protein was expressed and secreted into the culture medium, evidencing the functionality of the GP64 signal peptide. The rRBD migrated as a single band of about 30 kDa (theoretical mass without considering post-translational modification, 26.266 kDa) as judged by reducing SDS-PAGE (Figure 2A), and western blot developed with specific anti-His (Figure 2B) and anti-S antibodies (Figure 2C), demonstrating the identity of the protein. Under the non-reducing electrophoretic condition, two bands of rRBD appear showing the formation of disulfide-bound dimers (Figure 2D). The dimers are reversible upon the addition of a reductant in the SDS-PAGE sample buffer. Densitometry analysis showed that the presence of dimers is constant while monomer concentration increases gradually, achieving a maximal expression level at 4 dpi, where the dimer represents only 17.9% of the total rRBD expressed. Finally, rRBD was harvested at 4 dpi and its concentration in the cell culture, estimated by Bradford and gel densitometry assay (Supplementary Figure S2), was 25.6 ± 3.0 mg/L (5% of total protein). 

### 3.3. Purification of rRBD from Sf9 Cell Line

**Figure 3:**
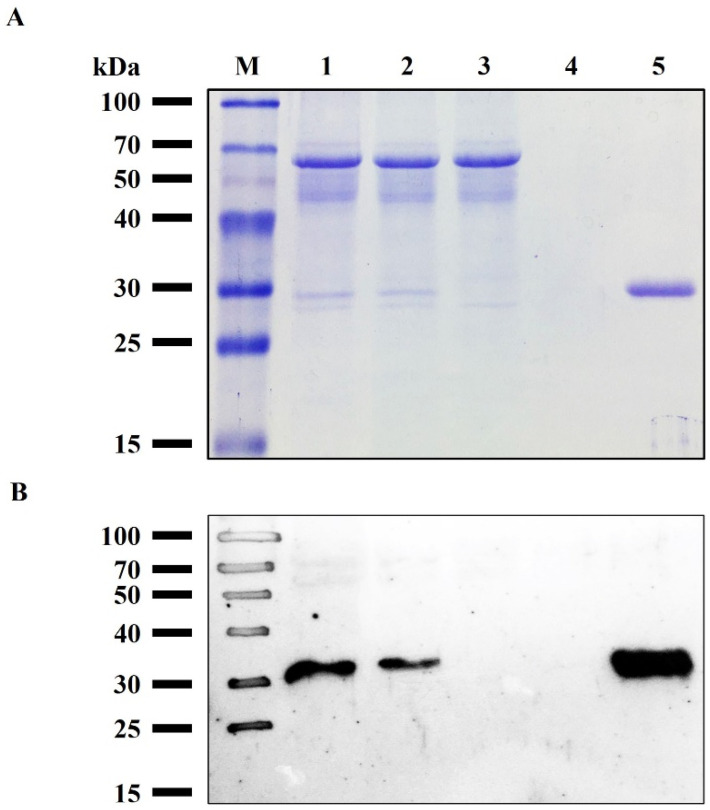
IMAC purification of rRBD. (**A**) SDS-PAGE analysis in the reducing condition of fraction collected during the purification process. (**B**) Western blot analysis of fraction collected during the purification process using anti-S antibody. Lanes: M, protein marker; 1, Sf9 cell expression supernatant; 2, diafiltrated sample (Input); 3, flow-through; 4, washing step (equilibration buffer with 80 mM imidazole); 5, IMAC fraction eluted by 500 mM imidazole.

**Figure 4:**
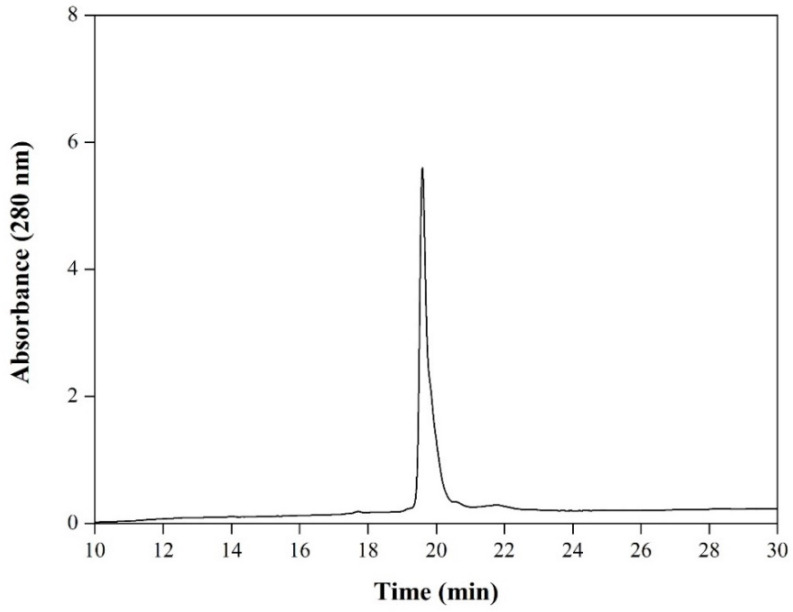
RP-HPLC analysis of rRBD purified by IMAC.

IMAC was performed to purify the rRBD from supernatant Sf9 cells. The cell-culture medium contained low fetal bovine serum (1% *v*/*v*), facilitating the purification step, and reducing the burden contaminants in the crude sample. The sample was conditioned by diafiltration with a recovery yield of 88% and loaded into the column. rRBD was adsorbed on the matrix with high affinity, confirmed previously by western blot. Most contaminating proteins, including EGFP, appeared in the flow-through (i.e., not adsorbed), and the addition of 20 mM imidazole to the equilibration buffer enhanced the purity level of rRBD. A washing step with 80 mM imidazole increased the final product purity as judged by the absorbance at 280 nm (Supplementary Figure S3). Finally, rRBD was desorbed only in the elution fraction containing 500 mM imidazole. Figure 3 shows the SDS-PAGE (Figure 3A) and western blot pattern (Figure 3B) of the purification fractions. Using the BEVS in Sf9 insect cell, combined with a novel chimeric promoter *polh-pSeL* in only one chromatographic step, it was possible to obtain 21.1 ± 3.7 mg/L culture of purified rRBD with a yield of 82%, which is the highest performance reported to date for Sf9 and High Five™ cells infected with recombinant baculovirus. The purity of the protein was higher than 95% as determined by RP-HPLC eluting in a single peak, indicating that it is a homogeneous sample (Figure 4). 

### 3.4. Characterization of the Purified RBD from Sf9 Insect Cell Culture

**Figure 5:**
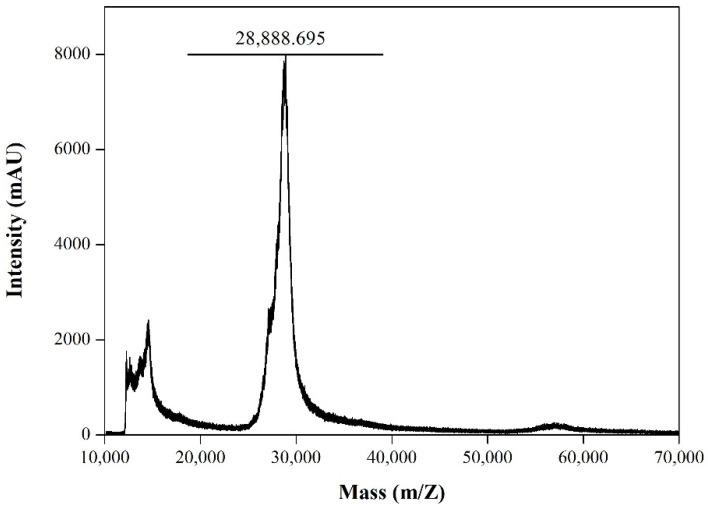
MALDI-TOF MS analysis of purified rRBD.

**Figure 6:**
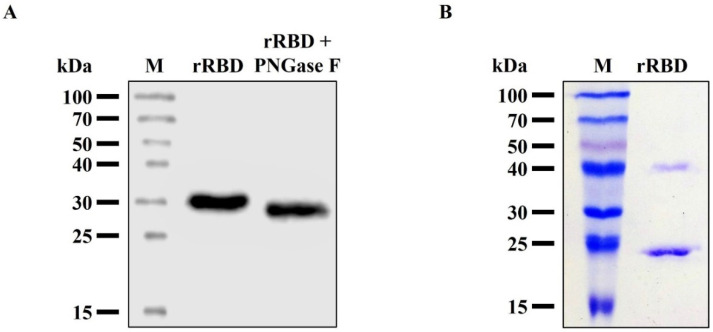
Characterization of purified rRBD. (**A**) rRBD glycosylation analysis. N-glycosidase F-mediated in-vitro deglycosylation of purified rRBD. Western blot in reducing condition developed with anti-S antibody. (**B**) Analysis of purified rRBD by SDS-PAGE in non-reducing condition. Lanes: M, protein marker; rRBD, purified rRBD; rRBD + PNGase. F, purified rRBD treated with N-glycosidase F.

The experimental mass (28.89 kDa) of rRBD was determined by the MALDI-TOF MS analysis (Figure 5). It has been reported that the SARS-CoV-2 RBD has two N-glycosylation sites (N331 and N343) that are fully glycosylated when expressed in heterologous expression systems [26]. To characterize the glycosylation status, the purified rRBD was treated with N-glycosidase F, an amidase that removes N-linked oligosaccharides from glycoprotein. After cleavage, we found a decrease in the molecular weight of the rRBD to about 26.0 kDa (Figure 6A). Thus, this evidence suggests that the N-glycosylation is responsible for the differential size between the treated and non-treated rRBD. Depending on the platform expression selected, the rRBD glycosylation degree varies, and this fact can strongly impact its ability to interact with its receptor ACE2 and specific human IgG [12]. In general, proteins expressed in insect cells exhibit paucimannose N-glycans, a less complex processed glycan than those produced in mammalian cells. Despite these differences, binding studies have shown that rRBD obtained from different mammalian and insect expression systems have comparable binding affinities to IgG against SARS-CoV-2 S, revealing, under certain conditions, a little higher binding ability of insect-derived rRBD [15,16]. It has been reported that the low molecular size of the insect type N-glycosylation produces a lower steric effect as compared to mammalian cells, constituting an economic and suitable platform to generate rRBD samples for basic studies, such as measuring the affinity with ligands or neutralizing antibodies [12,15]. Although the insect cell lines, in general, produce N-glycan, it should be noted that there is considerable difference between the N-glycan synthesized by High Five™ and that synthesized by Sf9 cells. High Five™ cells produce glycoproteins with core 1,3-linked fucose, which are highly immunogenic and make recombinant glycoprotein unacceptable for human use [27]. To address this problem, it is possible to use an alternative insect cell line such as Sf9 that produces 1,6-fucosylated N-glycan, similar to the glycan structure observed [28]. In this sense, the recombinant rRBD derived from infected Sf9 cells produced in the present study would be an appropriate antigen to be used for therapeutic purposes. 

**Figure 7:**
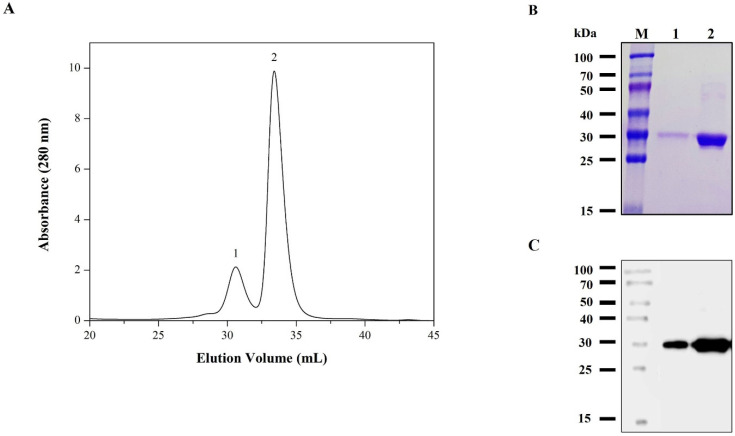
Size exclusion chromatography analysis of rRBD purified by IMAC. Chromatogram showing the elution profile of rRBD (**A**). The number indicates the peak containing rRBD dimer (1) and monomer (2). The peaks were analyzed by reducing SDS-PAGE (**B**) and western blot developed with specific anti-S antibody (**C**). Lanes: M, protein marker; 1, peak 1; 2, peak 2.

Furthermore, RBD has a total of 9 cysteine residues, eight of which form four pairs of disulfide bonds and unpaired residue (Cys538) close to the C-terminus responsible for the formation of oligomeric aggregate [15,29]. In addition, several hydrophobic patches have been identified on the surface of RBD that could promote noncovalent multimerization [30]. The analysis of purified rRBD by SDS-PAGE (Figure 6B) in the non-reducing condition showed the formation of a constant fraction of dimeric species, confirming what we had previously detected in the expression supernatant (Figure 2D). In addition, we evaluated the purified rRBD oligomeric state produced in our process by SEC (Figure 7A). The rRBD eluted as the main peak with an elution volume close to 33.3 mL and a minor one at 30.6 mL. The sample is homogeneous and mainly monomeric containing a low proportion (17%) of the dimer form, confirming what we had previously observed in the expression supernatant (Figure 2D). The purity of the monomeric RBD, after the SEC step, was over 98%, as judged by SDS-PAGE. The presence of rRBD in the two peaks was confirmed by SDS-PAGE (Figure 7B) and western blot (Figure 7C). The rRBD dimer formation has been reported in all expression systems used to date. Similar rRBD dimer levels to those found in insect cells were obtained in Chinese hamster ovary cells (16–23%) and Human embryonic kidney cells (12%). Additionally, in plant-based platforms, such as *Nicotiana benthamiana*, the presence of dimer is pronounced (75%) [15]. It has been reported that dimer content would not affect the immunochemical ability of rRBD to detect antibodies. However, dimer presence could affect the sample stability after long storage periods [31]. Although RBD dimer formation is a natural condition, through genetic engineering it is possible to increase the yield of the RBD monomeric form by removing or replacing unpaired Cys residue or certain amino acids located in the hydrophobic region to reduce the propensity to aggregate [15,30]. Alternatively, the multimeric protein can be separated by preparative SEC in order to obtain an even more homogeneous entity. Despite this, RBD dimer has been used to develop SARS-CoV-2 vaccine candidates because it is more immunogenic than the monomer [32,33].

### 3.5. Use of the RBD to Detect Anti-SARS-CoV-2 IgG in Serum Samples from COVID-19-Positive Patients

**Figure 8:**
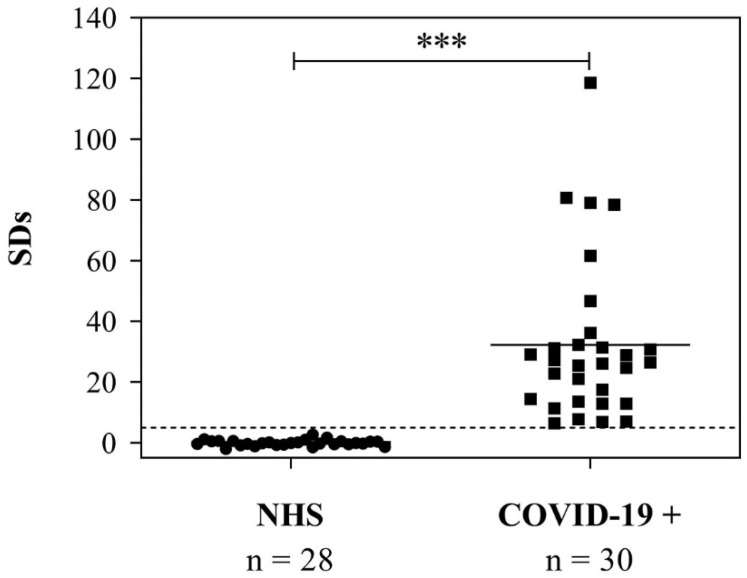
Immunoreactivity of rRBD produced in Sf9 cell line by b-ELISA. SARS-CoV-2 antibody test results obtained by b-ELISA from pre-pandemic samples (n = 28) and samples obtained from seropositive COVID-19 patients (n = 30). Results are expressed as SDs. The cut-off value (SDs > 5.0) is indicated by a dotted line and medians for each population are indicated as a full line (*** *p* < 0.0001, statistically significant). NHS: normal human sera. COVID-19+: seropositive COVID-19 patients.

Finally, we studied the immunochemical behavior of rRBD by evaluating its ability to detect antibodies in samples from COVID-19 patients by b-ELISA. To coat 96-well plates, 0.05 µg of purified rRBD per well was used. Figure 8 shows the distribution of signal obtained from 28 normal control sera and 30 IgG-positive COVID-19 patients. Median SDs for true negative samples were −0.09 (range: −1.98 to 2.73) and median SDs for true positive samples were 26.34 (range: 6.39 to 118.65). Results obtained from pre-pandemic control individuals differed significantly from those obtained from COVID-19-positive patients (*p* < 0.0001). An anti-S hyperimmune equine serum was also included as a positive control (SDs = 56.63). Previously, we had assayed the same ELISA design but using the full-length S protein expressed in *Rachiplusia nu* larvae as a coating antigen [25]. As expected, the median SDs obtained from COVID-19 patients were higher because the S complete version displayed a greater number of immunoreactive epitopes. Despite them, the rRBD derived from Sf9 cells was able to correctly discriminate between infected and uninfected individuals. This suggests that rRBD was immunoreactive to detect antibodies in serum samples. In addition, the kind of glycan structure typical of insects and the dimer proportion did not affect its immunochemical behavior and its ability to detect antibodies in patient samples. Moreover, the only purification step by IMAC was adequate to develop the assay. Therefore, the produced rRBD (17% dimeric form) is an appropriate standard for the development of diagnostic assays and 1 L of cell culture was enough to produce around 4400 plates of 96 wells.

## 4. Conclusions

In the present work, we developed for the first time a complete process for producing large amounts of the rRBD in Sf9 cells using the novel chimeric promoter *polh-pSeL*. We were able to achieve 21.1 ± 3.7 mg/L of purified RBD protein, which, to the best of our knowledge, is the highest yield reached using IC-BEVS. The results allow us to conclude that the platform is an interesting alternative engineering approach to produce rRBD as an antigen for the future test development for COVID-19 diagnosis.

## Data Availability

Not applicable.

## Supplementary Materials

The following supporting information can be downloaded at: https://www.mdpi.com/article/10.3390/v14122794/s1, Figure S1: Construction design of the pFBD-*polh-pSeL*-gprRBD vector; Figure S2: SDS-PAGE analysis and quantification of rRBD; Figure S3: Chromatogram of rRBD purification by IMAC.
