# Supplementary material for: Improved Expression of SARS-CoV-2 Spike RBD Using the Insect Cell-Baculovirus System
Source: Viruses. 2022 Dec 15;14(12):2794. doi: 10.3390/v14122794 (PMC9785345; doi:10.3390/v14122794)
Supplement: Supplementary file 1 [file viruses-14-02794-s001.zip › viruses-2075666-supplementary.pdf]

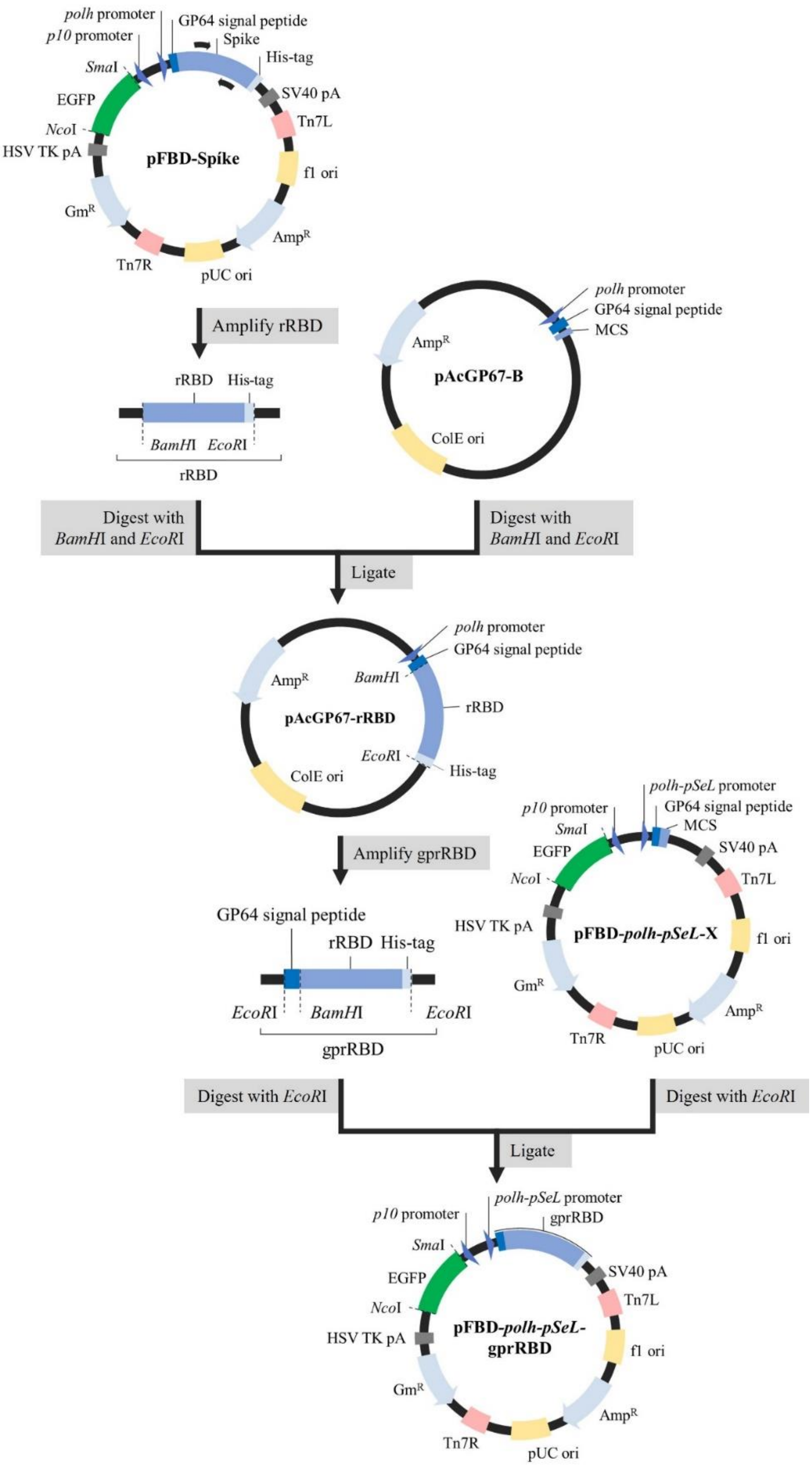

Figure S1. Construction design of the pFBD-*polh-pSeL*-gprRBD vector. **1**) The rRBD PCR product fused to the His-tag sequence was initially cloned into the *BamH*I and *Eco*RI sites of the pAcGP67-B vector. **2**) The gprRBD PCR product was cloned under the control of the *polh-pSeL* promoter into the *Eco*RI site of the pFBD-*polh-pSeL*-X vector to generate the pFBD-*polh-pSeL*-gprRBD vector. pFBD: pFastBac™ Dual Vector (Thermo Fisher Scientific); rRBD: recombinant SARS-CoV-2 spike receptor binding domain; gprRBD: rRBD fused in frame to the viral secretion signal GP64 and His-tag. Not to scale.

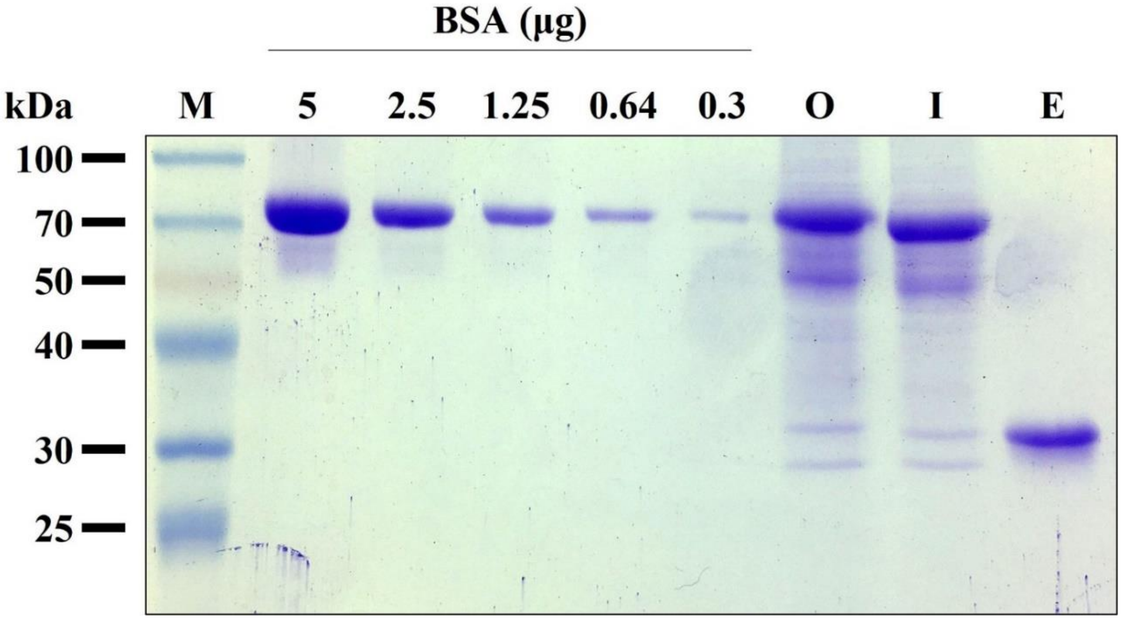

Figure S2. SDS-PAGE analysis and quantification of rRBD. Lanes: M, protein marker; O, Sf9 cell expression supernatant total protein extract; I, diafiltrated sample (Input); E1, IMAC fraction eluted by 500 mM imidazole.

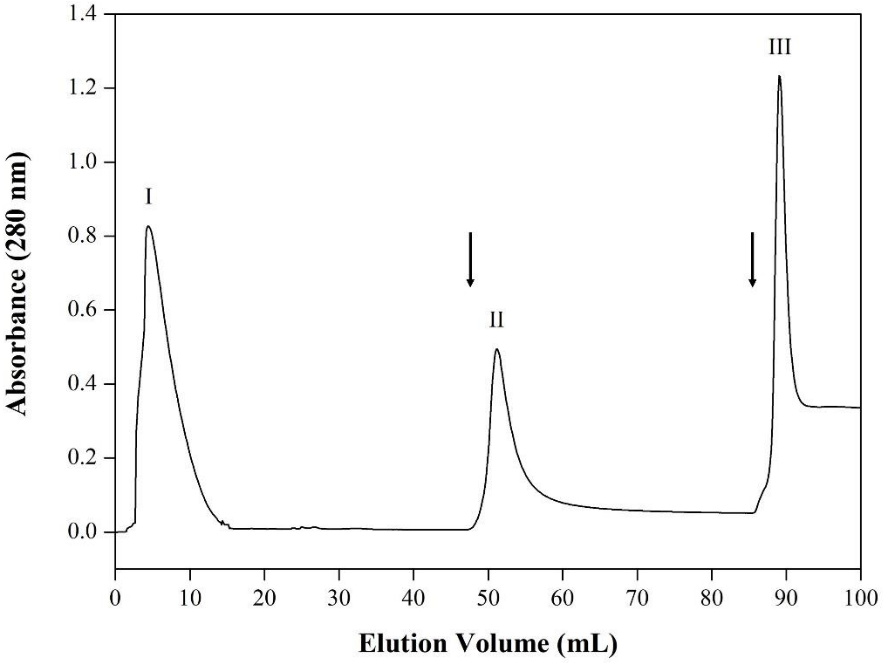

Figure S3. Chromatogram of rRBD purification by IMAC. The sample preconditioned in equilibration buffer (20 mM phosphate buffer pH 8.0, 300 mM NaCl, 20 mM imidazole) was loaded on a column packed with 3 mL Nuvia IMAC Ni-NTA Resin. Most contaminating proteins appeared in the pass-through (peak I). The column was washed with 80 mM imidazole equilibration buffer (peak II) and rRBD eluted with 500 mM imidazole (peak III). The absorbance at 280 nm were monitored. Arrows indicate the buffer change.
